# Rapid gist perception of meaningful real-life scenes: Exploring individual and gender differences in multiple categorization tasks

**DOI:** 10.1068/i0682

**Published:** 2015-01-06

**Authors:** Steven Vanmarcke, Johan Wagemans

**Affiliations:** Laboratory of Experimental Psychology, University of Leuven (KU Leuven), Leuven, Belgium, e-mail: steven.vanmarcke@ppw.kuleuven.be; Laboratory of Experimental Psychology, University of Leuven (KU Leuven), Leuven, Belgium, e-mail: Johan.Wagemans@psy.kuleuven.be

**Keywords:** ultrarapid categorization, rapid gist perception, Reverse Hierarchy Theory, individual differences, gender differences, social perception

## Abstract

In everyday life, we are generally able to dynamically understand and adapt to socially (ir)elevant encounters, and to make appropriate decisions about these. All of this requires an impressive ability to directly filter and obtain the most informative aspects of a complex visual scene. Such rapid gist perception can be assessed in multiple ways. In the ultrafast categorization paradigm developed by Simon Thorpe et al. (1996), participants get a clear categorization task in advance and succeed at detecting the target object of interest (animal) almost perfectly (even with 20 ms exposures). Since this pioneering work, follow-up studies consistently reported population-level reaction time differences on different categorization tasks, indicating a superordinate advantage (animal versus dog) and effects of perceptual similarity (animals versus vehicles) and object category size (natural versus animal versus dog). In this study, we replicated and extended these separate findings by using a systematic collection of different categorization tasks (varying in presentation time, task demands, and stimuli) and focusing on individual differences in terms of e.g., gender and intelligence. In addition to replicating the main findings from the literature, we find subtle, yet consistent gender differences (women faster than men).

## 1 Introduction

How quickly can we correctly determine what we see? What are the most important determinants to categorize a visual scene correctly? These and other questions motivated Thorpe, Fize, and Marlot (1996) to investigate ultrarapid object perception of complex natural images. In a predefined go/no-go categorization task with a briefly flashed (20 ms) visual scene, participants were able to detect the presence of exemplars of an object class (animal) nearly perfectly (the average proportion of correct responses was 94%). Further analysis revealed a concurrent, category-dependent differentiation in event-related potentials (ERPs) between go and no-go trials, primarily characterized by a frontal negativity following 150 ms after stimulus onset, for no-go trials only. These results revealed that visual processing as required by a predefined rapid categorization task can be done correctly in less than 150 ms. The perceptual demands for such an ultrarapid categorization task change by altering the specific, predetermined task required from the participants. Important to note is that object categorization can take place at different levels of abstraction within a hierarchical organization of semantic information (Rosch, Mervis, Gray, Johnson, & Boyes-Braem, 1976). The classic study by Rosch et al. (1976) identified the basic level as entry point for visual identification and categorization (e.g., “dog” rather than “animal” or “golden retriever”). Several recent studies with an ultrarapid categorization design similar to the design by Thorpe et al. (1996) contradicted these earlier results and interpretations by Rosch et al. (1976). Those recent studies indicated that participants are faster at detecting an animal/vehicle (superordinate object level) than a dog/bus (basic object level) in a complex visual image (Macé, Joubert, Nespoulous, & Fabre-Thorpe, 2009; Praβ, Grimsen, König, & Fahle, 2014). Similar findings (Kadar & Ben-Shahar, 2012; Rousselet, Joubert, & Fabre-Thorpe, 2005) were reported for scene gist categorizations (i.e., understanding the essential but broad meaning of a scene in a nutshell, e.g., as “street” or “beach”): Participants were faster at distinguishing between manmade/natural (superordinate scene level) than between sea/mountain (basic scene level). This consistent behavioral effect in ultrarapid categorization tasks is denoted as the superordinate advantage and seems robust for increased presentation times of the stimuli (Poncet & Fabre-Thorpe, 2014). Investigations into additional stimulus factors also indicated an influence of animacy (Crouzet, Joubert, Thorpe, & Fabre-Thorpe, 2012; Praβ et al., 2014), possibly due to overall differences in perceptual similarity between different categories (animals versus vehicles).

In order to replicate and extend these separate findings on ultrarapid categorization *[aim 1]*, a systematic collection of different ultrarapid categorization tasks was used in the current study. This collection could be subdivided into three main task paradigms: (1) the behavioral baseline, (2) the animal/vehicle, and (3) the social task. The first, behavioral baseline task (circle versus triangle), controls for possible individual or group-level differences in simple categorical decisions and motor responses. The main variables of interest in the second, animal/vehicle task, were Animacy, Level of categorization, and Goal. “Animacy” refers to the detection of either an animate (Dog/Animal/Natural) or an inanimate (Car/Vehicle/Artificial) object or scene class, while “Level of categorization” refers to the detection of either a basic (Dog/Car) or a superordinate object (Animal/Vehicle) or scene (Natural/Artificial) class. The third variable, “Goal.,” refers to the categorization of either a specific, salient object within an image (Dog/Car/Animal/Vehicle) or the scene (Natural/Artificial) itself (Kadar & Ben-Shahar, 2012; Macé et al., 2009; Praβ et al., 2014; Rousselet et al., 2005). The third, social, task paradigm focuses on the detection of an emotionally relevant (Positive social interaction) situation within the image, while also providing another scene (Indoor) level categorization task. The presentation time of the social task, which required an emotionally relevant judgment, was longer (83 ms) in comparison with the other categorization tasks (33 ms) because a pilot study indicated that deciphering a social interaction correctly requires more time (see also Wagner, Kelley, & Heatherton, 2011).

Within the current literature on ultrarapid categorization, almost no attention is devoted to the presence of individual or group-level differences within the selected set of participants. Nevertheless, most research in this tradition is based on population-level reaction time (RT) measures for which previous findings clearly indicate a possible influence of, e.g., gender (Der & Deary, 2006) and intelligence (Deary, Der, & Ford, 2001). The precise effects (directionality and strength) of these variables on behavioral performance in RT tasks appear to depend strongly on the exact task demands (Olson, Hooper, Collins, & Luciana, 2007). Due to the succession of different categorization tasks, problems with task switching could also have an influence on the behavioral performance of the participants (Ravizza & Carter, 2008). Previous physiological (Lang, Greenwald, Bradley, & Hamm, 1993), behavioral (Erwin et al., 1992), and neural (Kret & De Gelder, 2012; Whittle, Yücel, Yap, & Allen, 2011; Wrase et al., 2003) research would also predict gender differences on the assessment and detection of emotionally relevant stimuli such as those used in the social task. Concurrently, other recent studies (Auyeung et al., 2009; Baron-Cohen, 2004; Wheelwright et al., 2006) indicate gender differences on two main psychological dimensions: “empathizing” (E) and “systemizing” (S). The former dimension (E: men < women) is defined as the desire to identify someone's mental state and to act in accordance with this, while the latter (S: men > women) is seen as the desire to analyze and construct a system in terms of its underlying regularities. In the current study, we will specifically focus on possible individual and group-level differences *[aim 2]* by measuring (1) intelligence using a validated short (four subtests) version of the WAIS-III (Sattler, 2001; Wechsler, 1997), (2) executive functioning by means of the Behavior Rating Inventory of Executive Function for Adults (BRIEF-A) questionnaire (Roth, Isquith, & Gioia, 2005), and (3) the psychological dimensions Empathizing (E) and Systemizing (S) with EQ and SQ-R questionnaires (Wheelwright et al., 2006). In order to further capture the individual predictive value of these different descriptive measures (e.g., TIQ, EQ, SQ-R, and BRIEF-A) on the population-level performance in the ultrarapid categorization tasks (behavioral baseline, animal/vehicle, and social task), we also focused on calculating the appropriate bilateral correlations between these different measures [*aim 3*].

The preceding analysis of the current literature on ultrarapid categorization provides us with the necessary background to formulate the different goals for the current study. These aims were threefold: (1) a general replication of the most common findings with respect to ultrarapid categorization (e.g., superordinate advantage), (2) an investigation of the presence of any important individual or group-level differences in behavioral performance (e.g., gender) on the different categorization tasks, and (3) an examination of bivariate correlations between the different descriptive measures and their relation to the group-level performance on the categorization tasks.

## 2 Materials and methods

All participants were tested on three different ultrarapid categorization tasks, a short version of an intelligence test (WAIS-III) and several descriptive questionnaires (BRIEF-A, EQ, and SQ-R). Test order was randomized across participants. An overview of the full battery of tasks, tests, and questionnaires, as well as the test order, is provided in Appendix 1.

### 2.1 Subjects

A group of 48 typically developing adults (24 men, 24 women) with a mean age of 20.67 years old (*SD* = 2.08) were tested on the current test battery. All participants were students at the University of Leuven (KU Leuven) and had normal or corrected-to-normal vision. They were informed about the study and received course credits or payment for participation. The study was conducted in line with the ethical principles regarding research with human participants as specified in The Code of Ethics of the World Medical Association (Declaration of Helsinki). The study was approved by the Ethical Committee of the Faculty of Psychology and Educational Sciences (EC FPPW) of the University of Leuven (KU Leuven), and the participants provided written informed consent before starting the experiment.

### 2.2 Computer tasks

This section provides an overview of the different computer tasks with an ultrarapid categorization design completed by all participants. Subjects were seated at 57 cm from the calibrated (gamma corrected) computer monitor (resolution: 1920 × 1200; refresh rate: 60 Hz; type: Monitor DELL U2410) in a dimly lit room. The head position of the participants was stabilized by means of a head and chin rest during testing.

#### 2.2.1 Behavioral baseline

**Figure 1.**
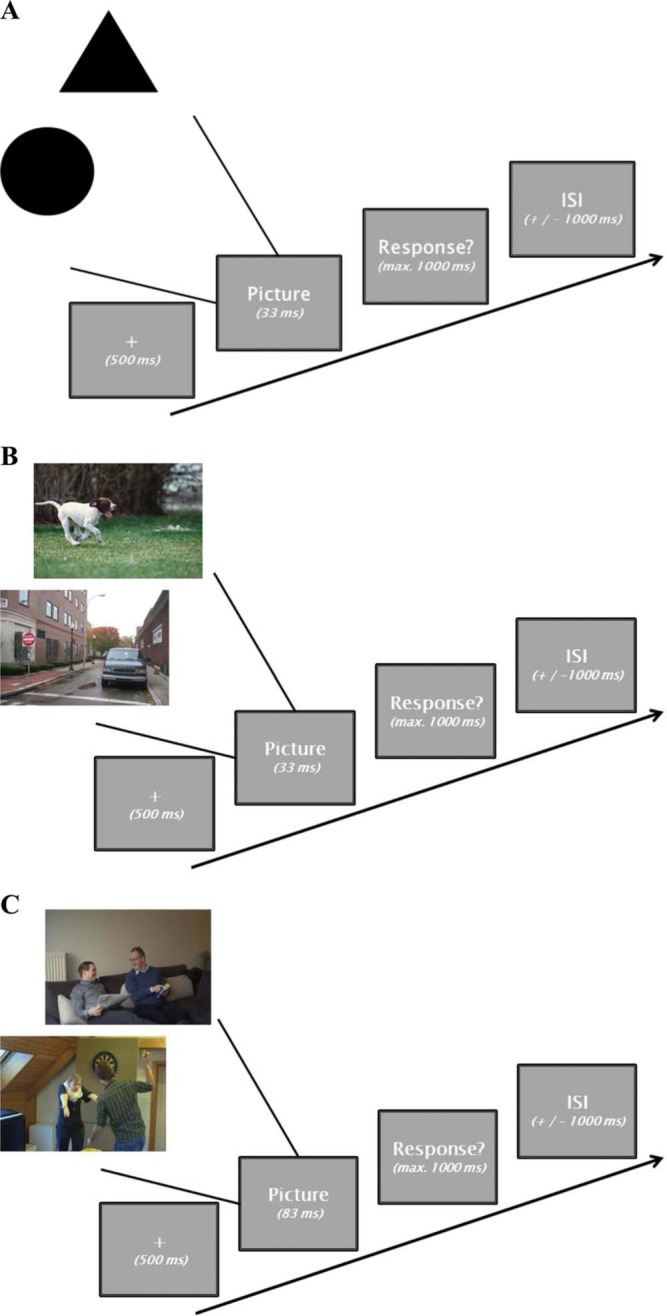
A graphical overview of the trial design of the different ultrarapid categorization tasks. The behavioral baseline (A) and animal/vehicle task (B) have the exact same layout with a presentation time of 33 ms. The social task (C) only differs in having a slightly longer PT of 83 ms.

To control for possible individual or group-level differences in simple categorization decision making and concurrent motor responding, a short control task (Figure 1A) was administered in which participants had to respond to the brief presentation of a geometrical figure (a black circle or triangle). A fixation cross (apparent size: 1 × 1° of visual angle) appeared for 500 ms, directly followed by a briefly flashed (33 ms) picture of either a black circle or triangle (average apparent size: 5 × 5°). Thereafter, participants got a 1000 ms response window to answer one of the following two questions: (1) Is there a triangle on the screen? or (2) Is there a circle on the screen?. Half of the participants (balanced for gender) got the former question, while the other half responded to the latter. If the presented stimulus contained the target, subjects had to press the space bar (go trial) as quickly as possible. If no target was present (no-go trial), participants had to wait for the next stimulus to appear. The intertrial interval (ITI) was randomized within a range of 1,000–1,500 ms. A total of 100 stimuli (50% targets; randomized order) were shown to each of the participants. Before onset of the actual experiment, participants got a brief practice session with visual trial-by-trial feedback (a green fixation cross was shown after each correct response, a red fixation cross after each incorrect response) on their performance to familiarize them with the specific design (eight stimuli; 50% targets). During the actual experiment, no feedback was provided.

#### 2.2.2 Animal/vehicle task

In the second rapid categorization task (Figure 1B), participants were asked to respond to the brief presentation of a meaningful color picture (apparent size: 18 × 12.5°). A fixation cross (apparent size: 1 × 1°) appeared for 500 ms, directly followed by the briefly flashed (33 ms) image. Next, subjects got a 1,000 ms response window to answer one of the following six questions: (1) *Is the scene artificial (manmade)?*, (2) *Is the scene natural?*, (3) *Is there a vehicle in the scene?*, (4) *Is there an animal in the scene?*, (5) *Is there a car in the scene?* or (6) *Is there a dog in the scene?*. The task was divided in six consecutive blocks, each consisting of 100 stimuli (50% targets; randomized order), in which a different question had to be answered each time. The order of the questions (blocks) was randomized across subjects and all participants completed all six blocks. If the presented stimulus contained the target, subjects had to press the space bar (go trial) as quickly as possible. If no target was present (no-go trial), participants had to wait for the next stimulus to appear. The ITI was randomized within a range of 1,000–1,500 ms. Before onset of the actual experiment, participants got a brief practice session with visual trial-by-trial feedback (a green fixation cross was shown after each correct response, a red fixation cross after each incorrect response) on their performance to familiarize them with the specific design (four stimuli per block; 50% targets). During the actual experiment, no feedback was provided.

#### 2.2.3 Social task

In the third rapid categorization task (Figure 1C), subjects had to respond to the brief presentation of a meaningful color picture (apparent size: 28 × 19°). A fixation cross (apparent size: 1 × 1°) appeared for 500 ms, directly followed by the briefly flashed (83 ms) image. Subjects got a 1,000 ms response window to answer one of the following two questions: (1) *Is the scene happening indoor?* or (2) *Is there a positive interaction (friendship) present in the scene?* The task was divided in two consecutive blocks, each consisting of the same 100 stimuli (50% targets; randomized order). The order of the questions (blocks) was randomized across subjects and all 48 participants completed both blocks. The ITI was randomized within a range of 1,000–1,500 ms. Before onset of the actual experiment, participants got a brief practice session with visual trial-by-trial feedback (a green fixation cross was shown after each correct response, a red fixation cross after each incorrect response) on their performance to familiarize them with the specific design (eight stimuli per block; 50% targets). During the actual experiment, no feedback was provided.

### 2.3 Intelligence test

To estimate intellectual ability, a shortened version of the Wechsler Adult Intelligence Scale, Third Edition (WAIS-III; Wechsler, 1997) was administered. This version consists of four subtests: Vocabulary, Similarities, Picture Completion, and Block Design (Sattler, 2001). The former two subtests give a general indication of Verbal Intelligence (VIQ), while the latter two measure Performance Intelligence (PIQ). The average of both provided an assessment of Full-Scale Intelligence (FSIQ).

### 2.4 Questionnaires

This section provides an overview of the different self-report questionnaires, which were completed by all participants.

#### 2.4.1 BRIEF-A

The Behavior Rating Inventory of Executive Function for Adults (BRIEF-A) questionnaire (Roth et al., 2005) contains 75 items, and yields an overall score of executive functioning comprising two separate indices: Behavioral regulation and Metacognition. While the former is based on four different subscales (Inhibition, Shift, Emotional control, and Self-control), the latter is built of five separate scales (Initiate, Working Memory, Plan/Organize, Organization of materials, and Task monitor). Norm scores are calculated for each of the clinical scales, indices, and their overall composite. Higher scores reflect more problems within the specific domain of measurement.

#### 2.4.2 EQ

The Empathizing Questionnaire (EQ) consists of 40 empathy items and 20 filler/control items (Baron-Cohen & Wheelwright, 2004). It is designed to measure the observer's emotional response to the affective state of another person. The questionnaire provides a single overall Empathizing score. Higher scores reflect a stronger emotional response toward others' affective states.

#### 2.4.3 SQ-R

The Systemizing Questionnaire-Reversed (SQ-R) consists of 75 systemizing items (Wheelwright et al., 2006). Systemizing is defined as the drive to analyze, understand, predict, control, and construct rule-based systems. The questionnaire provides a single overall Systemizing score. Higher scores reflect a higher tendency to analytically evaluate and control your surroundings.

### 2.5 Stimuli

**Figure 2.**
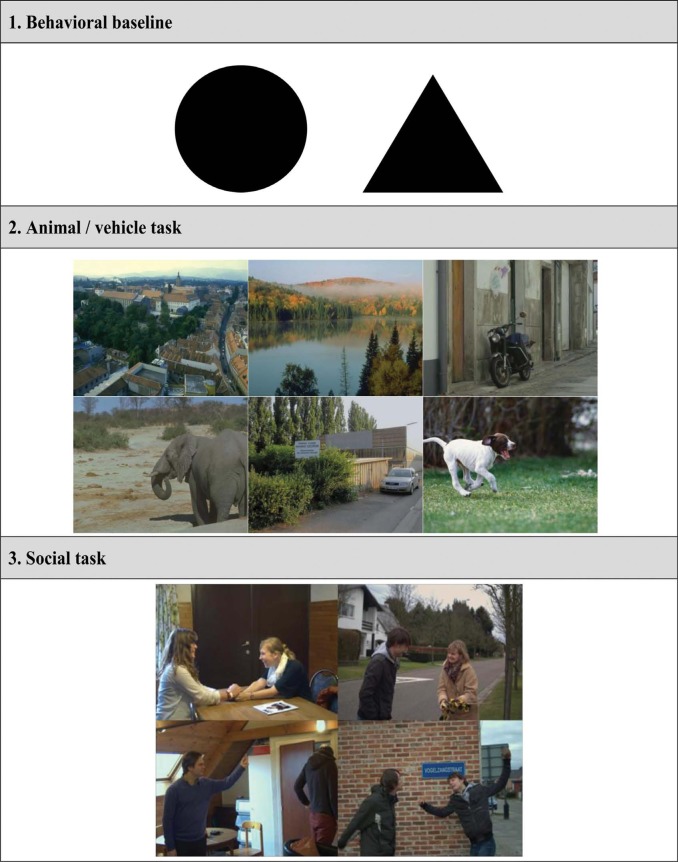
A general overview of the type of images used within the scope of the different ultrarapid categorization tasks. The complete picture set is made available online on http://gestaltrevision.be/en/resources/supplementary-material.

This section provides an overview of the stimuli used in the ultrarapid categorization tasks. The images used in the short practice sessions preceding the different experiments were always different from those in the actual testing phase. Some examples of the used stimuli in the tasks are shown in Figure 2. All images can be found on http://gestaltrevision.be/en/resources/supplementary-material.

#### 2.5.1 Behavioral baseline

A total set of 108 black, geometrical figures were created using the open-source software library PsychoPy, which is written in Python (Peirce, 2008). These images were either triangles (*n* = 54) or circles (*n* = 54) of different sizes (average size: 185 × 185 pixels; [min max] size: [136 234] × [136 234] pixels) and presented only once (either as target or nontarget) during the experiment or practice session. The mean luminance of the figures on the screen was 1–3 cd/m^2^;.

#### 2.5.2 Animal/vehicle task

A total set of 624 color images (650 × 460 pixels) was used for this task. All of these were selected (by unanimous consensus between several lab members including the first author) as being unambiguously artificial (*n* = 104), natural (*n* = 104), vehicle (*n* = 104), animal (*n* = 104), car (*n* = 104), or dog (*n* = 104) pictures. They were presented only once (either as target or nontarget) during the experiment or practice session. In line with previous research (e.g., Macé et al., 2009; Praβ et al., 2014), targets and nontargets of the same level of categorization were used in each stimulus category: at superordinate level, both for ultrarapid scene (Natural/Artificial) and object categorization and at basic level only for ultrarapid object (Dog/Car) categorization. To make this more explicit: (1) in the artificial (natural) category, natural (artificial) stimuli were used as nontargets; (2) for the animal (vehicle) category, vehicle (animal) and car (dog) stimuli were used as nontargets, and (3) in the car (dog) category, vehicle (animal) and dog (car) stimuli were used as nontargets. In each of the different image categories, a wide variety of possible scenes were selected. In order to avoid low-level confounds eliciting behavioral differences between stimulus categories (Joubert, Rousselet, Fabre-Thorpe, & Fize, 2009; Wichmann, Braun, & Gegenfurtner, 2006), each of the selected images was set to the same global luminance and Root Mean Square (RMS) contrast (corresponding to a luminance distribution, within the RGB color spectrum, with a mean of [123.98; 128.00; 109.31] and a standard deviation of [24.13; 25.00; 25.02]) by computing the average luminance and RMS contrast across all images. The mean luminance of the images on the screen was 63–68 cd/m².

#### 2.5.3 Social task

A total set of 108 color images (1024 × 688 pixels) was used for this task. These images were captured by the first author with a professional camera (Fujifilm FinePix S5 Pro) and shot in different indoor (bedroom, living room, kitchen, etc.) and outdoor (garden, forest, meadow, etc.) settings. In each of these images, two people were depicted, who either interacted in a positive manner, or in a negative or neutral (absence of interaction) manner (treated as one single category). To evaluate the effectiveness of the affective manipulation, 10 separate participants rated the entire picture set on a scale from 1 (very negative) to 9 (very positive). These ratings were then used to select the final stimulus set. More precisely, each selected image depicting a positive interaction had a mean individual rating score above 7, while the average rating value across all positive pictures was 7.80 (*SD* = .42). Each selected image depicting a negative/neutral interaction had a mean individual rating score below 5, with an overall score of 3.66 (*SD* = .76) across all negative/neutral pictures. This final set can be divided further into four different categories: (1) indoor and positive interaction (*n* = 27), (2) outdoor and positive interaction (*n* = 27), (3) indoor and negative/neutral interaction (*n* = 27), and (4) outdoor and negative/neutral interaction (*n* = 27). In both blocks, the same stimuli were used but the categorization criterion of the stimuli shifted from scene state (indoor versus outdoor) to social state (positive versus negative/neutral). The first two categories were the targets (the last two categories the nontargets) when a positive interaction needed to be detected, while the first and third category became the targets (second and fourth category the nontargets) when participants had to detect an indoor setting. In each of the different image categories, a wide variety of possible social interactions (positive, negative, neutral) were present. In order to avoid low-level confounds eliciting behavioral differences between stimulus categories (Joubert et al., 2009; Wichmann et al., 2006), each of the selected images was set to the same global luminance and Root Mean Square (RMS) contrast (corresponding to a luminance distribution, within the RGB color spectrum, with a mean of [116.39; 110.00; 98.44] and a standard deviation of [25.61; 25.00; 21.88]) by computing the average luminance and RMS contrast across all images. The mean luminance of the images on the screen was 33–38 cd/m².

### 2.6 Analysis

#### 2.6.1 Computer tasks: Within and between individual differences

To evaluate the ultrarapid categorization tasks, both go response reaction time (RT) and accuracy (correct/incorrect) per trial were taken into account as the dependent variables (DV) in a General Linear Modeling (GLM) approach (McCullagh, 1984). The latter was done for each of the computer tasks separately, in order to fully account for possible differences between subjects and across trials. In each of the different random intercepts (logistic), regression analysis deviance values were calculated for the different models based on a maximum likelihood estimation (Aitkin, 1999) of either RT or accuracy on each of the given tasks. By assessing the drop in deviance (DiD) together with the Akaike Information Criterion (AIC; Akaike, 1973) and Bayesian Information Criterion (BIC; Schwarz, 1978) values (a general overview can be found in Appendix 2), the final model was selected. After model selection, the individual predictive value of each selected parameter was tested using (1) Welch's *t* test with Satterthwaite approximation for the denominator degrees of freedom (McArdle, 1987) in the random intercepts regression analysis for RT and (2) Wald *Z* tests (Wald, 1943) in the random intercepts logistic regression (link function is *logit(p)* = *log (p* / *(1-p))*) analysis for accuracy. Participant was always regarded as random intercept and descriptive measures (e.g., TIQ, BRIEF-A and Gender) were tested as possible covariates in each of these analyses. In the behavioral baseline, only Task (Circle versus Triangle) was regarded as a fixed effect in the final model. In the animal/vehicle task, Gender (Man versus Woman), Time (Moment of testing), Level of categorization (Basic versus Superordinate), Animacy (Inanimate versus Animate), and Goal (Object versus Scene) were kept as fixed effects in the final model. In the social task, Gender (Man versus Woman) and State (Scene versus Social) were used as fixed effects in the final model. All outcomes were obtained by using the lme4 package (Bates, 2005) of the statistical software program R 3.1.1 (R core team, 2013).

#### 2.6.2 Descriptive measures

The scores on the different (sub)scales of the intelligence test and the questionnaires were calculated as recommended by the test developers. On each of the tests separately, a one-way ANOVA with Gender (Man versus Woman) as a between-subjects factor was conducted.

#### 2.6.3 Population-level differences

In order to further capture possible between-subject population-level differences within the dataset, median go response reaction time (RT) and mean performance (accuracy) values were also calculated. The latter was operationalized by means of the sensitivity (*d′*) measure (Macmillan & Creelman, 1991). This monotonic function provides an indication of the performance for each observer, by combining the Hit (*H*) rate (proportion correctly judged go trials) with the False Alarm (FA) rate (proportion incorrectly judged no go trials) into a single standardized score: *d′* = *Z[H] – Z[*FA*]*. Within this framework, *Z* corresponds to the inverse of the normal distribution function. For each of the computer tasks, a linear regression analysis (Gelman & Hill, 2006) was conducted, in which we mainly focused on the individual predictive value of the different descriptive measures (e.g., TIQ, EQ, SQ-R, and BRIEF-A) on (1) the average performance (RT and sensitivity) in the ultrarapid categorization tasks and (2) the difference scores between the different testing blocks within a certain task (e.g., median RT “animal”—median RT “artificial”). All outcomes were obtained by using the statistical software program R 3.1.1 (R core team, 2013).

Finally, we also calculated the bilateral Pearson correlation coefficients (Pearson's *r*) between the different descriptive measures. Another explorative analysis was conducted using partial bilateral Pearson correlation coefficients (Pearson's *r*) controlling explicitly for gender, age, and/or total IQ. In all these correlation analyses, a Bonferroni correction for multiple comparisons was applied (Bland & Altman, 1995).

## 3 Results

### 3.1 Computer tasks

#### 3.1.1 Behavioral baseline

The final model (see Table 1 for parameter estimates and 95% confidence intervals) for both RT and performance provided a significant random intercept (RT: *t_47.97_* = 39.11; *p* < .001 | Accuracy: *Z* = 10.09; *p* < .001), but did not indicate any evidence for differences in task RT or accuracy due to whether people had to report either a circle or a triangle. This conclusion was supported by the nonsignificant effect of adding the fixed (between-subject) factor Task (RT: *t_47.97_* = −.32; *p* = .75 | Accuracy: *Z* = −1.01; *p* = 0.31) to the model. Other descriptive variables (e.g., Total IQ, BRIEF-A, and Gender) taken into account as covariates provided similar nonsignificant outcomes. The mean expected reaction time and accuracy given the random intercepts model was therefore similar across all participants (irrespective of, e.g., Gender; RT = 386 ms | Accuracy (*%*) = .99), and similar to the actual observed outcomes for men (RT = 388 ms | Accuracy (*%*) = .99) and women (RT = 383 ms | Accuracy (*%*) = .99). This indicates that the simple decision and execution of the motor response did not elicit any meaningful differences between the different participants.

**Table 1.** Overview of the parameter estimates for the behavioral baseline task for both (a) the random intercepts regression analysis on the RT output and (b) the random intercepts logistic regression analysis on the accuracy data.

RT			
Parameter	Estimate (SE)	*p*-value	95% confidence interval
Intercept	.386 (.010)	< .001	[.365; .405]
Task	−.004 (.014)	.749	[−.033; .024]

Accuracy			
Parameter	Estimate (SE)	*p*-value	95% confidence interval
Intercept	5.574 (.553)	< .001	[4.491; 6.657]
Task	−.646 (.646)	.313	[−1.902; .609]

#### 3.1.2 Animal/vehicle task

**Figure 3.**
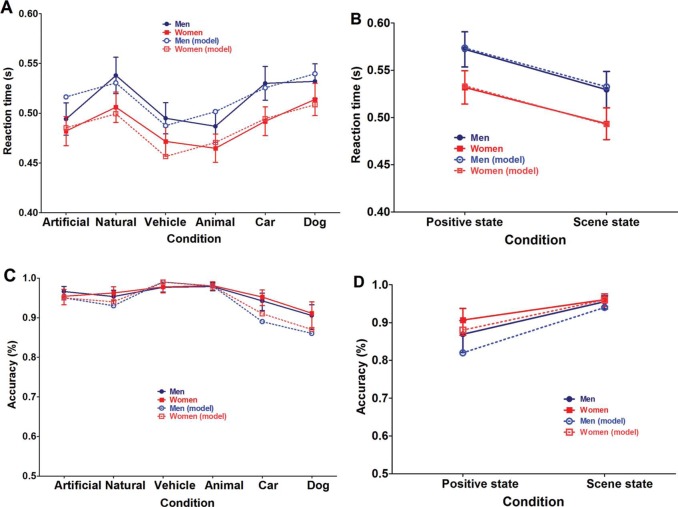
Overview of reaction time (A) and accuracy (C) outcomes in the ultrarapid categorization animal/vehicle task. The data are represented as the mean performance across participants, with error bars depicting the standard error of the mean (SEM). For accuracy, mean and SEM were calculated based on the logistic transformation of the values and then retransformed into percentage correct (%) data. A similar overview is provided for the reaction time (B) and accuracy (D) outcomes in the ultrarapid categorization social task. Men are always depicted in blue, women in red. The dotted lines depict the mean expected RT (A, B) or accuracy (C, D) as fitted by the final model for both men (light blue) and women (light red) based on (1) a random intercepts regression analysis for RT and (2) a random intercepts logistic regression analysis for accuracy. The latter fit was averaged (for both RT and accuracy) over time.

The final model (see Table 2 for parameter estimates and 95% confidence intervals) for both RT and accuracy (see Figure 3A and C) provided a significant random intercept (RT: *t_59.64_* = 44.21; *p* < .001 | Accuracy: *Z* = 15.24; *p* < .001). The analysis furthermore provided a clear *replication of the superordinate effect [Aim 1]* (e.g., Macé et al., 2009; Praβ et al., 2014) due to the significant impact of the fixed (within-subjects) factor Level of Categorization (RT: *t_47.18_* = −10.27; *p* < .001 | Accuracy: *Z* = 12.72; *p* < .001) on outcome prediction. It also yielded an interesting finding regarding the processing of scene gist perception versus object perception, namely a significantly faster and more accurate categorization of “Object” versus “Scene” information. The latter was exemplified by the significant effect of the fixed (within-subjects) factor Goal on predicting task RT and accuracy (RT: *t_46.45_* = 8.47; *p* < .001 | Accuracy: *Z* = −6.60; *p* < .001). The conducted random intercept regression analysis also indicated that people were faster and more accurate at detecting Inanimate versus Animate information as exemplified by the significant effect of the fixed (within-subjects) factor Animacy (RT: *t_45.96_* = 3.61; *p* < .001 | Accuracy: *Z* = −3.19; *p* < .01) on outcome prediction. The moment of testing (tested by adding the fixed (within-subject) factor Time (RT: *t_26.96_* = 1.12; *p* = .27 | Accuracy: *Z* = −1.70; *p* = .09) did not lead to a significantly better prediction of the dependent variables. This indicated that the randomized sequence of test blocks did not elicit any detrimental/learning effects on RT or accuracy.

**Table 2.** Overview of the parameter estimates for the Animal/vehicle task for both (a) the random intercepts regression analysis on the RT output and (b) the random intercepts logistic regression analysis on the accuracy data.

RT			
Parameter	Estimate (SE)	*p*-value	95% confidence interval
Intercept	.519 (.012)	< .001	[.495; .543]
Level of Categorization	−.038 (.004)	< .001	[−.046; −.031]
Goal	.029 (.003)	< .001	[.022; .036]
Animacy	.014 (.004)	< .001	[.006; .022]
Gender	−0.031 (.013)	.023	[−.058; −.005]
Time	.002 (.002)	.273	[−.002; .005]

Accuracy			
Parameter	Estimate (SE)	*p*−value	95% confidence interval
Intercept	2.073 (.136)	< .001	[1.806; 2.339]
Level of Categorization	1.414 (.111)	< .001	[1.196; 1.632]
Goal	−.759 (.115)	< .001	[−.984; −.534]
Animacy	−.270 (.085)	< .01	[−.436; −.104]
Gender	.092 (.108)	.392	[−.118; .302]
Time	−.053 (.031)	.089	[−.113; .008]

**Figure 4.**
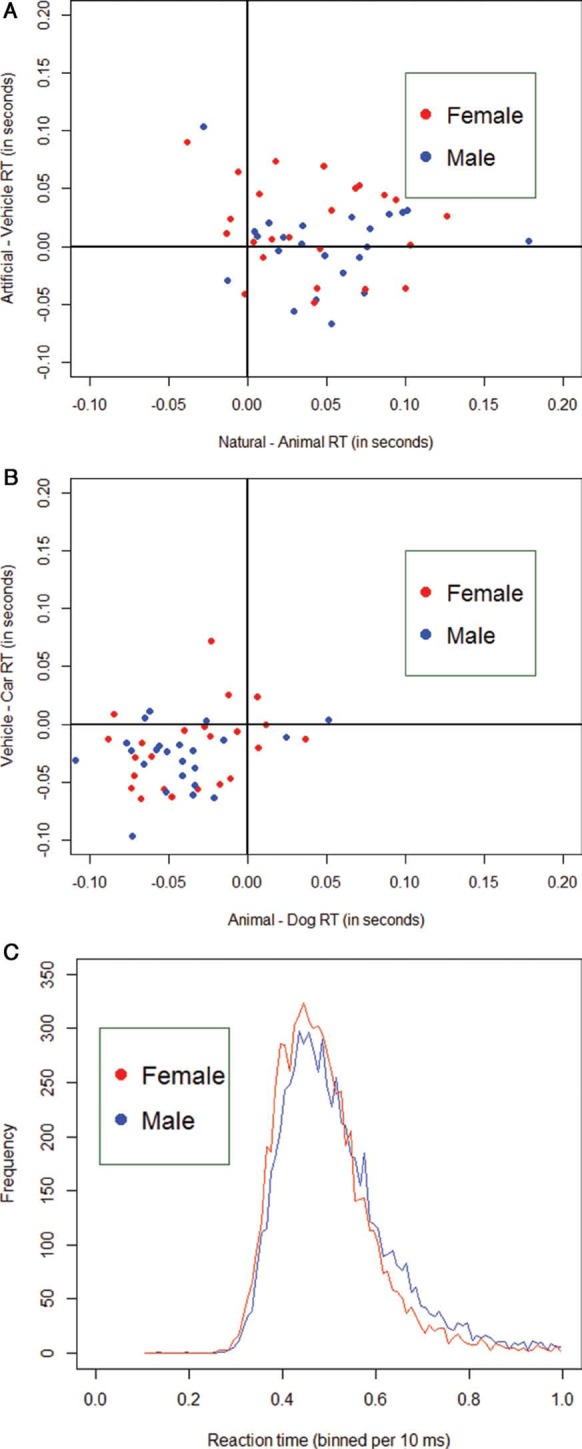
Visualization of the data by means of scatterplots. In (A), we placed the individual subjects' median RT difference scores of the Artificial versus the Vehicle condition on the abscissa and the difference scores of the Natural versus the Animal RT scores on the ordinate axis. Men are always depicted in blue, women in red. For both axes, the largest part of the RT distribution lies above the origin (positive difference scores). This would indicate that people are generally faster at detecting an object (e.g., Vehicle or Animal) than detecting the scene gist (e.g., Artificial or Natural) in ultrarapid categorization. In (B), we placed the individual subjects' median RT difference scores of the Vehicle versus the Car condition on the abscissa and the difference scores of the Animal versus the Dog RT scores on the ordinate axis. For both axes, the largest part of the RT distribution lies below the origin (negative difference scores). This would indicate that people are generally faster at detecting a superordinate object (e.g., Vehicle or Animal) than detecting a basic-level object (e.g., Car or Dog). Furthermore, both scatterplots indicate differences in the specific RT distribution for men or women. The latter finding is also exemplified in (C), in which all RT data in the Animal/Vehicle task are binned per 10 ms and plotted with respect to frequency. The female RT distribution (red) peeks slightly earlier than the male RT distribution (blue) and has a lighter right tale.

Other descriptive variables (e.g., Total IQ, BRIEF-A, SRS-A, and Age) taken into account as covariates did not provide a significant improvement in predicting RT or accuracy. But further exploratory analysis of the observed fixed effects indicated the possible presence of Gender differences (Figure 4). Specifically checking for these *group-level Gender differences [Aim 2]*, we did find significantly faster RT for women in comparison to men. This could be modeled by adding the fixed (between-subjects) factor Gender (RT: *t_46.01_* = −2.29; *p* = .02) as a predictor of RT outcomes. With regard to the accuracy values, adding Gender (Accuracy: *Z* = .86; *p* = .39) to the model did not lead to any significant improvements in prediction.

In order to assess the specific location of these gender effects in the RT distributions, we also analyzed the quartiles of the individual RT distributions within the framework of the final model (see Figure 3A). No quartile-specific differences were observed for the fixed effects (e.g., Goal, Level of Categorization and Animacy) reported in the general analysis (on the entire RT distribution). Only the group-level Gender differences were sensitive to the actual speed of responding. While there were no clear Gender differences present in the fasted response times (1st quartile: *t_48_* = −1.69; *p* = .10 | 2nd quartile: *t_48_* = −1.82; *p* = .08), these did become more strongly present when response times became longer (3rd quartile: *t_48_* = −2.23; *p* = .03 | 4th quartile: *t_48_* = −2.06; *p* = .04).

#### 3.1.3 Social task

The final model (see Table 3 for parameter estimates and 95% confidence intervals) for both RT and accuracy (see Figure 3B and D) provided a significant random intercept (RT: *t_55.05_* = 42.65; *p* < .001 | Accuracy: *Z* = 9.81; *p* < .001). More interestingly, the analysis also indicated clear differences between judging whether a “positive interaction” was present and judging whether the scene was happening “indoor.” More specifically, perceiving a social state took significantly more time and was less accurate than identifying the scene state. The latter finding was observed by adding the fixed (within-subjects) factor State (RT: *t_47.87_* = −5.96; *p* < .001 | Accuracy: *Z* = 11.71; *p* < .001) as a predictor of each of both dependent variables. This effect is in accordance with the more complex nature of judging social information (Wagner et al., 2011). Adding the fixed (within-subjects) factor Time (RT: *t_48.04_* = 1.37; *p* = .18 | Accuracy: *Z* = −1.65; *p* = .10) to the model, did not elicit any significant differences in estimating RT or accuracy outcome values. This indicated that the randomized test order of both blocks did not elicit any strong detrimental/learning effects on RT nor on accuracy.

**Table 3.** Overview of the parameter estimates for the Social task for both (a) the random intercepts regression analysis on the RT output and (b) the random intercepts logistic regression analysis on the accuracy data. All outcomes were obtained by using the lme4 package (Bates, 2005) of the statistical software program R 3.1.1.

RT			
Parameter	Estimate (SE)	*p*-value	95% confidence interval
Intercept	0.569 (.0133)	< .001	[.542; .596]
State	−0.041 (.007)	< .001	[−.055; −.027]
Gender	−0.040 (.018)	.030	[−.077; −.004]
Time	0.009 (.007)	.178	[-.004; .023]

Accuracy			
Parameter	Estimate (SE)	*p*-value	95% confidence interval
Intercept	1.221 (.124)	< .001	[.977; 1.465]
State	1.154 (.099)	< .001	[.961; 1.347]
Gender	.332 (.155)	.031	[.030; .635]
Time	−.154 (.093)	.098	[−.336; .029]

**Figure 5.**
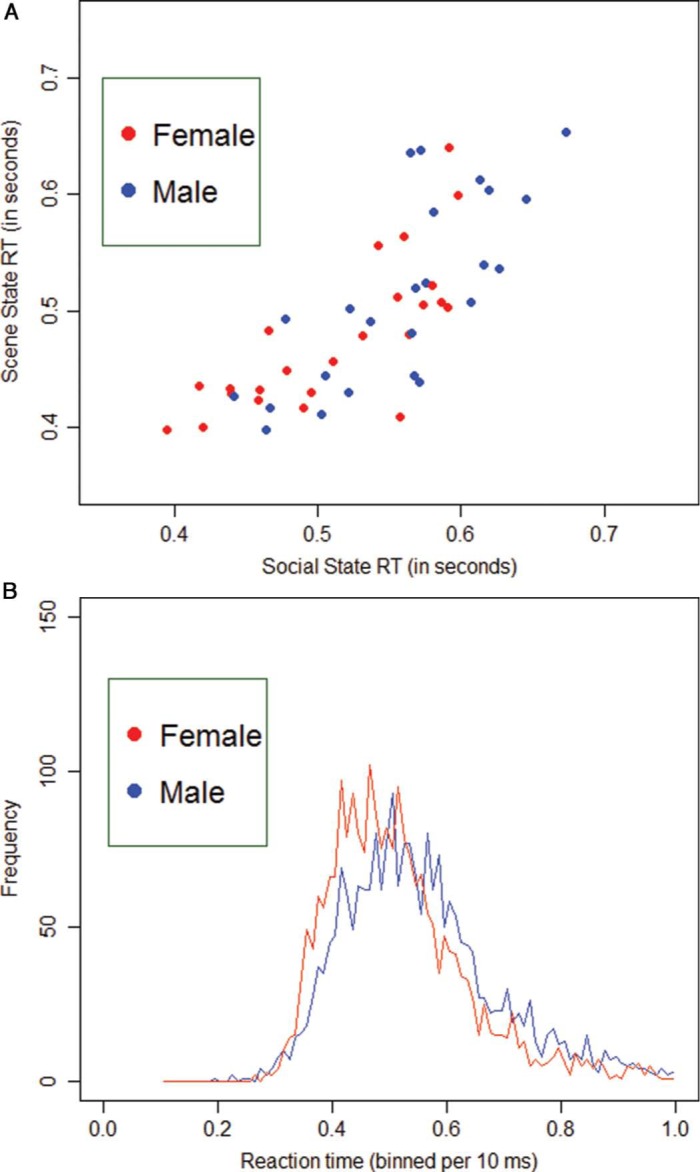
Visualization of the data by means of a scatterplot in (A). The individual subjects' median RT scores in the Social State (*Is there a positive interaction (friendship) present in the scene?*) are placed on the abscissa and those of the Scene State (*Is the scene happening indoor?*) on the ordinate axis. Men are always depicted in blue, women in red. Two things are noticeable from this graphical depiction: (1) people are generally faster at categorization of the Scene State in comparison with the Social State and (2) men seem to have larger RTs (on both tasks) in comparison to women. Similar conclusions can be drawn from (B), in which all RT data in the Social task are binned per 10 ms and plotted with respect to frequency. The female RT distribution (red) peaks clearly earlier than the male RT distribution (blue) and has a slightly lighter right tail.

Other descriptive variables (e.g., Total IQ, BRIEF-A, and Age) taken into account as covariates did not provide a significant improvement in predicting RT or accuracy. Specifically checking for *group-level Gender differences [Aim 2]*, we did find significantly faster RT and higher accuracy values for women in comparison to men in judging both social and scene state (Figure 5). This was observed by adding the fixed (between-subjects) factor Gender (RT: *t_47.86_* = −2.24; *p* = .03 | Accuracy: *Z* = 2.15; *p* = .03) as a predictor of each of both dependent variables. The latter finding is in line with previous research indicating gender-specific responses with respect to emotionally relevant stimuli (Erwin et al., 1992; Kret & De Gelder, 2012; Lang et al., 1993; Wrase et al., 2003).

In order to assess the specific location of these gender effects in the RT distributions, we also analyzed the quartiles of the individual RT distributions within the framework of the final model (see Figure 3B). No quartile-specific differences were observed for the fixed effects (e.g., State and Time) reported in the general analysis (on the entire RT distribution). But, similar to the Animal/Vehicle task, the group-level Gender differences became more pronounced with larger response times. While there were no clear Gender differences present in the first quartile of response times (*t_47.94_* = −1.88; *p* = .07), these did become more strongly present when response times became longer (2nd quartile: *t_47.90_* = −2.26; *p* = .03 | 3rd quartile: *t_47.92_* = −2.31; *p* = .03 | 4th quartile: *t_47.69_* = −2.38; *p* = .02).

### 3.2 Descriptive measures

An overview of the average scores across all participants (with standard deviation) and the gender-specific group-level outcomes for each of the described tests (and subscales) can be found in Table 4.

**Table 4.** An overview of the average scores, on the different descriptive measures and their subscales, both across participants and at the gender-specific group level. The standard deviation (SD) is always provided between brackets.

Variable	Men	Woman	All
Number of participants	24	24	48
Age	20.67 (2.08)	20.92 (1.50)	20.79 (1.80)
EQ	39.25 (10.38)	47.58 (9.64)	43.42 (10.77)
SQ-R	60.29 (16.76)	48.29 (19.24)	54.29 (18.85)
Full-Scale IQ	108.31 (7.04)	106.77 (7.18)	107.54 (7.07)
Verbal IQ	109.08 (7.17)	112.08 (6.88)	110.58 (7.12)
Performal IQ	107.54 (11.91)	101.46 (10.72)	104.50 (11.62)
BRIEF-A (Overall)	56.50 (7.82)	56.92 (7.39)	56.71 (7.53)
BRIEF-A (Behavioral regulation)	50.17 (6.62)	55.13 (8.30)	52.65 (7.84)
BRIEF-A (Metacognition)	60.58 (9.80)	57.71 (7.96)	59.15 (8.95)
BRIEF-A (Inhibition)	56.52 (10.03)	57.33 (55.71)	55.71 (9.11)
BRIEF-A (Shift)	53.23 (10.02)	51.29 (10.80)	55.17 (8.98)
BRIEF-A (Emotional control)	51.46 (11.06)	45.71 (6.97)	57.21 (11.50)
BRIEF-A (Self-control)	49.21 (8.37)	50.00 (8.29)	48.42 (8.56)
BRIEF-A (Initiate)	60.00 (10.36)	61.92 (11.43)	58.08 (9.01)
BRIEF-A (Working memory)	56.94 (9.45)	58.00 (10.86)	55.88 (7.89)
BRIEF-A (Plan/Organize)	57.73 (59.08)	59.08 (10.61)	56.38 (9.37)
BRIEF-A (Organization of materials)	56.45 (10.89)	55.74 (11.25)	57.13 (10.73)
BRIEF-A (Task monitor)	57.77 (11.16)	62.25 (10.17)	53.29 (10.45)

#### 3.2.1 BRIEF-A

Scores fell within the normal range of the norm group both for the overall and for the separate dimensional scores. With respect to the summarizing index scores, gender differences were only found on behavioral regulation (*F_1;46_* = 5.24; *p* = .027). Although both gender groups still scored within the normal range, women had significantly higher outcomes on this index score. When turning to the smaller subscales of the questionnaire, significant differences were also present on emotional control (*F_1;46_* = 17.55; *p* < .001) (women better than men) and task monitoring (*F_1;46_* = 9.06; *p* = .004) (women worse than men).

#### 3.2.2 EQ

Scores on Empathizing were similar to those attained in the large sample set on healthy adult students by Wheelwright and colleagues (Wheelwright et al., 2006). Women obtained significantly higher Empathizing scores than men on this questionnaire (*F_1;46_* = 8.31; *p* = .006).

#### 3.2.3 SQ-R

Scores on Systemizing were similar to those attained in the large sample set on healthy adult students by Wheelwright and colleagues (Wheelwright et al., 2006). Women obtained significantly lower Systemizing scores than men on this questionnaire (*F_1;46_* = 5.31; *p* = .026). The findings of significant differences on both EQ and SQ-R were in line with the gender-sensitive expectations of the EMB theory (Auyeung et al., 2009; Baron-Cohen, 2004; Baron-Cohen & Wheelwright, 2004).

#### 3.2.4 WAIS-III

Scores were generally 0.50−1.00 standard deviation above the average population performance. This was in accordance with the educational background of the participant set (all students at the University of Leuven). No significant gender differences were present.

### 3.3 Population-level differences

This section provides a brief overview of (1) the conducted population-level regression analysis predicting the population-level outcome performance (median RT and sensitivity) on the different computer tasks and (2) the calculated bilateral Pearson correlation coefficients between the different descriptive measures in order *to investigate the descriptive measures and their relation to the ultrarapid categorization tasks [Aim 3].*

#### 3.3.1 Descriptive measures and computer tasks

For each of the computer tasks, a linear regression analysis (Gelman & Hill, 2006) was conducted, in which we mainly focused on the individual predictive value of the different descriptive measures (e.g., TIQ, EQ, SQ-R, and BRIEF-A) on (1) the average performance (median RT and sensitivity) in the ultrarapid categorization tasks and (2) the difference scores between the different testing blocks within a certain task (e.g., median RT “animal”— median RT “artificial”). For none of the separate computer tasks, we found a strong significant contribution (all: *p* > .01) of adding one of the descriptive predictors (e.g., TIQ, Age, EQ, SQ-R, and BRIEF-A) in explaining the variance in the dependent variable and none of the found effects were consistent across RT and sensitivity. The general lack of consistency leads us to conclude that none of the descriptive variables had a strong predictive value in explaining the data. This was in agreement with the random intercepts (logistic) regression analysis provided in the previous section.

#### 3.3.2 Descriptive measures only

The correlation matrix (Table 5) indicated a negative correlation between SQ-R and BRIEF-A (*r* = −.47; *p* = .001), higher Systemizing correlating with less vulnerability to problems in executive functions, and remained significant with a Bonferonni corrected level of significance (*p* = .005). Given that scores on both questionnaires remain within the normal range, it could be argued that this could be due to systematically analyzing a situation based on a rule-based problem solving approach and correctly regulating and planning of executive functions (Best, Miller, & Naglieri, 2011).

**Table 5.** Overview of the correlation matrix between the different descriptive measures with an indication of those effects which were significant with a (Bonferonni) corrected level of significance (*p* = .005).

	SQ-R	EQ	IQ	BRIEF - A
SQ-R	1			
EQ	−0.14	1		
IQ	0.11	−0.36	1	
BRIEF-A	−0.47*	−0.14	−0.05	1

*Note.* Overview of the used abbreviations: SQ-R = Systemizing Quotient-Reversed, EQ = Empathizing Quotient, RC (BB) = Rapid categorization (Behavioral Baseline), WAIS-III = Wechsler Adult Intelligence Scale-III, BRIEF-A = Inventory of Executive Function for Adults.

* (*p* < .005).

## 4 Discussion

### 4.1 Overview research questions

We summarize the different outcomes of this ultrarapid categorization study based on the three predetermined goals for the current study.

#### 4.1.1 [Aim 1] Replication of the most common findings with respect to ultrarapid categorization

A clear replication of the superordinate effect was observed in the animal/vehicle task (e.g., the significant effect of Level of categorization in predicting both RT and accuracy). Similar to previous ultrarapid categorization studies (Macé et al., 2009; Praβ et al., 2014; Rousselet et al., 2005), the more abstract (superordinate) object information is available earlier than more concrete (basic) level representations of its subcategories. A recent study indicated that this advantage is present, even in cases of longer (500 ms) stimulus presentation times (Poncet & Fabre-Thorpe, 2014). One way to explain these findings is by the time-course and task-dependent predictions of the feed-forward parallel distributed processing (PDP) theory (Rogers & McClelland, 2004; Rogers & Patterson, 2007). It states that semantic, superordinate information will initially be activated before basic-level information because it applies to a wider range of semantically related items. This translates into the observed superordinate effect in the ultrarapid categorization literature (Macé et al., 2009). We also observed a general main effect of Animacy (in predicting both RT and accuracy) in the animal/vehicle task. Inanimate objects or scenes were categorized faster and with fewer mistakes. Previous findings on the effect of animacy in ultrarapid categorization were rather ambiguous: one study (Praβ et al., 2014) reported a similar inanimate advantage, others found a small but significant animate advantage (Crouzet et al., 2012; Crouzet, Kirchner, & Thorpe, 2010), while yet another study did not even find an effect of animacy (VanRullen & Thorpe, 2001) on performance. Possible explanations for these seemingly incoherent findings are differences in (1) stimulus set (e.g., lack of perceptual similarity), (2) task demands (e.g., different levels of categorization used), and (3) presentation times (e.g., between 33 and 83 ms). One way to explain the inanimate advantage found here might be explained by the correlated feature theory (Tyler et al., 2004). This theory suggests that animate objects or scenes contain more common similar features (e.g., has legs, can see,…) than their nonliving counterparts. The animal/vehicle task indicated that processing the scene level (i.e., detecting an animate, “natural” scene) was done more slowly (RT) and less accurately than processing the object level (i.e., spotting an “animal” in the image) (significant effect of Goal in predicting both outcome variables). Similar findings were reported in a recent study by Crouzet et al. (2012). They explained their results through a rapid feed-forward process based on the hardwired extraction of specific, complex animal features evolved from an evolutionary advantage for processing animate information (New, Cosmides, & Tooby, 2007). Given that we also observed a faster RT and better performance on “vehicle” versus “natural” scenes, this explanation does not seem to capture all aspects of our findings. Furthermore, similar differences were found with respect to the “artificial” scene detection. An alternative explanation (Davenport & Potter, 2004; Joubert, Rousselet, Fize, & Fabre-Thorpe, 2007; Mack & Palmeri, 2010) stipulates the existence of an advantage based on the presence of a salient object within an object-consistent background (object-scene consistency advantage). This is suggested to result from the inherent influence certain objects have on the global scene statistics diagnostic for scene categorization. The stimuli used for the current study were selected in such a manner that no salient objects (such as vehicle or animal) were present in the “artificial” and “natural” scenes, while this was (by definition) the case in the “vehicle” and “animal” pictures. In the latter image sets, these objects always appeared on a consistent background (“artificial” and “natural,” resp.). But to correctly interpret and quantify these observed differences in both animacy and object/scene detection, future research will have to focus on (1) benchmarking the specific information available in each of the selected image sets (VanRullen, 2011) and (2) specifically focusing on identifying the low-level image properties associated within the used stimulus set (Joubert et al., 2009; Wichmann et al., 2006). Such follow-up research will have to infer the exact importance of these low-level physical properties (e.g., shape, orientation, and overall complexity) in explaining the observed differences in this ultra-rapid categorization study.

#### 4.1.2 [Aim 2] Group-level Gender differences

Gender differences in RT and accuracy (the significant effect of Gender in predicting both outcome variables) were both observed in the animal/vehicle and in the social task. To our knowledge, this is the first study on ultrarapid categorization which explicitly reports on these group-level differences. This can be explained by several elements: (1) The participant set (48 participants) used in this study is markedly larger than those reported in the literature (i.e., 12–20 participants), which could have led to general, yet subtle, tendencies going unnoticed previously (Baguley, 2004); (2) none of the previous ultrarapid categorization tasks used a task where a judgment of a socially relevant interaction was required; and (3) the systematic experimental protocol provided a diverse collection of test material which could reveal contextual consistencies across different (in)animate levels of categorization.

With respect to the ultrarapid animal/vehicle categorization task, women were generally faster and more accurate than men in judging whether an object or scene was present or not. Further analysis showed that this Gender effect was mainly based on differences in the slower responses (although the trends were in the same direction for the faster responses). The latter indicates that the RT distribution of women in ultrarapid categorization tasks have lighter right tails in comparison with those of men (Ratcliff, 1993). This finding, combined with a small shift of the entire female RT distribution toward the left (Figure 4C), characterizes the gender differences reported on this task. These cannot be accounted for by gender-specific task switching problems (Ravizza & Carter, 2008) following the succession of multiple testing blocks. There are three different arguments to support this claim: (1) the Time variable did not elicit a significant improvement in predicting RT or accuracy, (2) no gender differences were found on the executive functioning (BRIEF-A) questionnaire, and (3) the test order of the different stimulus blocks was completely randomized across participants. Possible other explanations for these gender-specific findings could relate to differences in either (1) the ability to rapidly extract the important global (high-level) elements of the presented stimulus in order to make a correct categorization decision (Bacon-Macé, Macé, Fabre-Thorpe, & Thorpe, 2005; Mack & Palmeri, 2011; Schyns & Oliva, 1994; Thorpe et al., 1996) or (2) the processing of category-specific low-level elements of the presented scenes (Joubert et al., 2009; Wichmann et al., 2006). Future, more focused, follow-up research on the exact stimulus properties of the object/scene categories will be necessary in order to answer these remaining questions. In the ultrarapid social categorization task, women were generally better and faster at detecting a social or scene state (significant effect of Gender in predicting both outcome variables) than men. With regard to the scene state (*Is the scene happening indoor?*), which is a superordinate categorization task at scene-level similar to the natural/artificial categorization, these findings could be regarded as similar to those in the scene categorization (artificial or natural) of the animal/vehicle task. With regard to the social state (*Is there a positive interaction (friendship) present in the scene?*), these gender differences in rapidly extracting scene information (e.g., indoor versus outdoor) from the stimulus might not be the main reason for the given findings (significant effect of State in predicting both outcome variables). Although this was the first time that stimuli presenting complex social interactions were used within an ultrarapid categorization framework, these outcomes were consistent with previous findings in the broader literature (Erwin et al., 1992; Lang et al., 1993; Kret & De Gelder, 2012; Whittle et al., 2011; Wrase et al., 2003). A previous affective priming study (Donges, Kersting, & Suslow, 2012) with short emotional prime presentations (33 ms) provided a similar female advantage in processing positive (happy) facial information. These gender differences in the emotional understanding of interpersonal interactions also coincide with the observed gender-specific variation in Empathizing and Systemizing (both in this study and in the literature) (Erwin et al., 1992; Kret & De Gelder, 2012; Wrase et al., 2003). They are therefore also consistent with previous findings indicating that men have more trouble understanding and acting upon socially relevant information (Auyeung et al., 2009).

#### 4.1.3 [Aim 3] Population-level differences (descriptive measures)

The general scores on the different descriptive measures (WAIS-III, BRIEF-A, SQ-R, and EQ) were well within the normal range for the tested participant set. Correlations between these tests revealed a correlation between higher Systemizing scores and a less pronounced vulnerability for problems with executive functioning. This could be explained by the development of a better problem-solving approach with higher Systemizing scores, as long as these do not exceed a certain threshold (Best et al., 2011). None of these correlations were regulated by gender. No consistent correlations between the descriptive measures and the performance measures (RT and *d′*) were found.
